# Verification of recall criteria for masses detected on ultrasound breast cancer screening

**DOI:** 10.1007/s10396-017-0778-5

**Published:** 2017-02-25

**Authors:** Kanako Ban, Hiroko Tsunoda, Sakiko Suzuki, Rie Takaki, Kyouko Sasaki, Minako Nakagawa

**Affiliations:** 1 Tokyo Health Service Association, Tokyo, Japan; 2 Research Group for Breast Cancer Screening of the Japan Association of Breast and Thyroid Sonology, Tokyo, Japan; 3 St Luke’s International Hospital, Tokyo, Japan; 4 Wellness Tenjin Clinic, Fukuoka, Japan; 5 Iwate Health Service Association, Morioka, Japan; 6 Okayama Health Foundation, Okayama, Japan

**Keywords:** Recall criteria, Breast cancer screening, Breast ultrasound, Ultrasonographic screening

## Abstract

**Purpose:**

Mammography is the only modality for breast cancer screening demonstrated to reduce the mortality rate. However, ultrasonographic screening is already being widely performed as opportunistic screening in Japan. The recall criteria for masses are very important as quality controls. The purpose of this study was to verify these criteria at multiple institutions.

**Methods:**

Screening was performed by five institutions in various regions in Japan. The total number of cases screened at all five institutions was 10,519.

**Results:**

The findings that could be concluded to be benign were a cystic pattern and three features of a solid pattern. The cystic pattern was noted in 6512 cases, typical fibroadenoma in 1483 cases, and typical complicated cyst in 70 cases. Only three of these 8065 cases were cancers, so the negative predictive value was 99.9%. The solid pattern with obvious malignant features, i.e., masses with an echogenic halo and/or interruption of the interface and masses with multiple echogenic foci, were noted in 33 cases. Twenty of the 33 cases were malignancy, resulting in a positive predictive value of 66.7%.

**Conclusion:**

Although some parts of the criteria should be considered further for verification and revision, the current recall criteria are mostly valid.

## Introduction

Mammography is the only modality of breast cancer screening demonstrated to reduce the mortality rate, but its detection ability is limited in premenopausal women with dense breasts, for which ultrasonography has been attracting attention. It has been demonstrated in a randomized controlled trial conducted in Japan [1] that many invasive cancer cases can be detected by adding ultrasonography to mammography, but its mortality rate-reducing effect has not been clarified, and slightly longer observation of the course is necessary for its analysis. Accordingly, evidence is still insufficient to introduce ultrasonography into population-based screening, but ultrasonographic screening is already widely performed in opportunistic screening in Japan, and the recall criteria are very important as a part of its quality control. The recall criteria for masses detected on screening described in the 3rd edition of the Guidelines for Breast Ultrasound Diagnosis [the Japan Association of Breast and Thyroid Sonology (JABTS)] were published in English on 16 February 2016 [2]. The recall criteria used for ultrasonographic screening were prepared based on consensus among experts. The criteria had previously been verified at only a single institution [3]. Thus, we tried to verify it based on data collected at five institutions in various regions in Japan.

## Materials and methods

The subjects and screening method at each institution are shown in Table 1. Screening performed by the five institutions partially included population-based screening, but many cases were examined in opportunistic screening.

**Table 1:** The subjects and screening method at each institution

Institutions	Subjects	Period	Number of cases	Methods	Equipments
1 Tokyo Health Service Association	Participant of J-START(Women in their 40 s)	Apr. 2009–Mar.2012	3005 cases 3348 findings	Static image	Toshiba Aplio400 Toshiba Xario XG probe 12L5
2 St Luke’s International Hospital	Examinee of opportunistic screening	Jan.–Jun 2011	1727 cases 5870 findings	Static image	Hitachi EUB-7500 probe linear7-13 MHz
3 Wellness Tenjin Clinic	Examinee of opportunistic screening	Apr. 2012–Mar. 2013	1283 cases 2117 findings	Static image	Toshiba SSA-790A Toshiba T-US400A probes PLT-1204BT, PLT-1204AT, PLT-805AT, PLT-704SBT
4 Iwate Health service Association	Participant of J-START(Women in their 40 s)	Apr. 2008–Mar. 2012	734 cases 1025 findings	Static image and movie	Toshiba Viamo(SSA 640A), probe PLT-805AT Toshiba Nemio(SSA 550A), probe PLM-805AT Toshiba Xario(SSA 660A), probe PLT-805AT Tochiba Aplio50(SSA 700A), probe PLT-805AT ALOKA SSD-4000, probe UST-5546
5 Okayama Health Foundation	Participant of J-START(Women in their 40 s) and Examinee of opportunistic screening	Sep. 2007–Mar. 2009	436 cases 236 1findings	Static image	Hitachi EUB 7500(EUP L-65) Hitachi Avius(EUP L74M) TOSHIBA Viamo(PLT-1204BT)

* J-START[2] (Japan Strategic Anti Cancer Randomized trial)

The total number of cases screened at all five institutions was 10,519. Ultrasonography was performed by ultrasonographers who had received passing certification (grade A or B) in the breast ultrasonography training course and test held by JABTS or The Central Organization on Quality Assurance of Breast Cancer Screening (COQABCS). All cases were interpreted using static images by physicians certificated (grade A or B) by COQABCS. One institute used both static images and movies for interpretation.

The findings were classified as categories 1–5, with categories 3–5 subjected to further examination (Table 2). The number of Category 2 cases was 9898. Six hundred twenty-one cases were assessed as Category 3 or higher and required further examination, and the recall rate was 5.9%. Breast cancer was detected in 58 cases evaluated as requiring further examination, whereas cancer was detected in four cases (0.4%) assessed as requiring no further examination, and the positive predictive value was 9.3%.

**Table 2:** The category classification (JABTS)

Assessment of possibility for malignancy
Category 1: negative
Category 2: benign or abnormal findings that further examination is not necessary
Category 3: benign but malignancy not ruled out
Category 4: suspicious abnormality
Category 5: highly suggestive malignancy

**Fig. 1:**
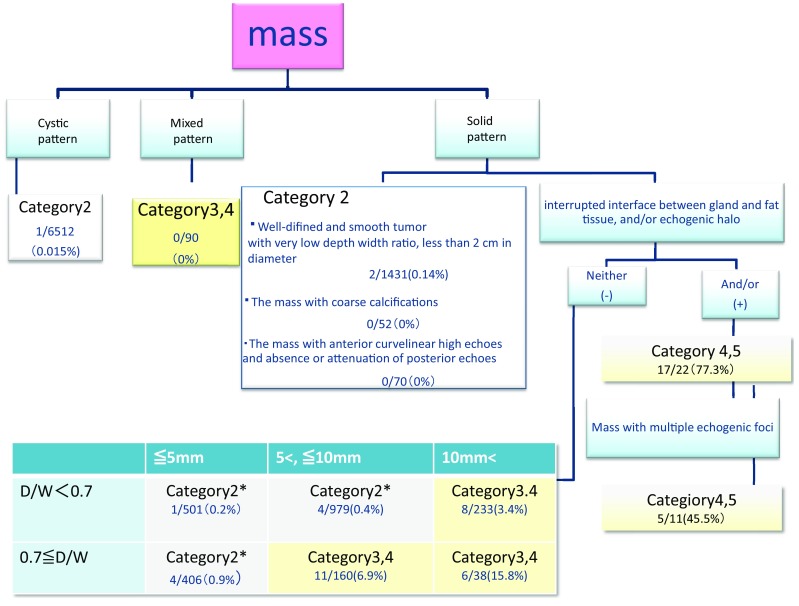
Malignant cases/number of findings (percentage)

The screening flow chart shown in Fig. 1 is presented in the Guidelines for Breast Ultrasound Diagnosis of JABTS [1]. The final result of benignity or malignancy was confirmed in each box of the flow chart, and the cancer discovery rate and positive predictive value were calculated. Benignity was assessed by cytology or histological examination. When biopsy was not performed, the absence of malignant findings was confirmed by 2-year or longer course observation.

## Results

Following the diagnostic tree of the recall criteria for masses, the masses were classified into cystic, mixed, and solid patterns. The results are reported by these classifications, dividing the solid-pattern masses into those with obviously benign and malignant findings and multiple echogenic foci, and cases to be finally assessed based on the maximum diameter and depth/width (D/W) ratio. The category, total number, and number of masses finally assessed as malignant are presented by feature in Table 3. In addition, the number of cases with malignant features and the rate of malignant cases are summarized in the box of the diagnostic tree in Fig. 1.

**Table 3:** The category, total number and number of masses finally assessed as malignant

Institutions		1	2	3	4	5	total
Cystic pattern	Category 2	1457	3968 (1)	532	No data	555	6512 (1)
Mixed pattern (<0.5 cm)	Category 2	0	0	3	0	0	3
Mixed pattern (≥0.5 cm)	Category 3	3	37	25 (1*)	10	15	90 (1*)
Well-defined and smooth tumor with very low depth width ratio, less than 2 cm in diameter	Category 2	85	1102	92	125 (2)	27	1431 (2)
The mass with coarse calcifications	Category 2	8	21	8	8	7	52
The mass with anterior curvilinear high echoes and absence or attenuation of posterior echoes	Category 2	No data	47	15	No data	8	70
Interrupted interface between gland and fat tissue, and/or echogenic halo	Category 4, 5	4 (4)	8 (6)	5 (3)	4 (3)	1 (1)	22 (17)
Mass with multiple echogenic foci	Category 4, 5	1 (0)	1 (1)	6 (1)	3 (3)	0 (0)	11 (5)
<5 mm (DW < 0.7)	Category 2	7	29	381 (1)	2	81	501 (1)
<5 mm (DW < 0.7)*shape irregularity	Category 3	1	0	0	0	0	0
<5 mm (DW ≥ 0.7)	Category 2	4	66	242	0	85	397
<5 mm (DW ≥ 0.7)*shape irregularity	Category 3	0	0	1 (1)	1 (1)	7 (2)	9 (4)
5–10 mm (DW < 0.7)	Category 2	111	43 (1)	598	8	169	929 (1)
5–10 mm (DW < 0.7)*shape irregularity	Category 3	6 (1)	34 (1)	0	8	7 (1)	50 (3)
5–10 mm (DW ≥ 0.7)	Category 2	2	0	0	0	0	2
5–10 mm (DW ≥ 0.7)	Category 3	18 (5)	61 (2)	50 (1)	8 (1)	23 (2)	160 (11)
≥10 mm (DW < 0.7)	Category 2	1	0	0	0	0	1
≥10 mm (DW < 0.7)	Category 3	10	51 (2)	151 (4)	8 (2)	12	232 (8)
≥10 mm (DW < ≥ 0.7)	Category 3	7 (3)	13	8	5 (1)	5 (2)	38 (6)

### Cystic pattern

**Fig. 2:**
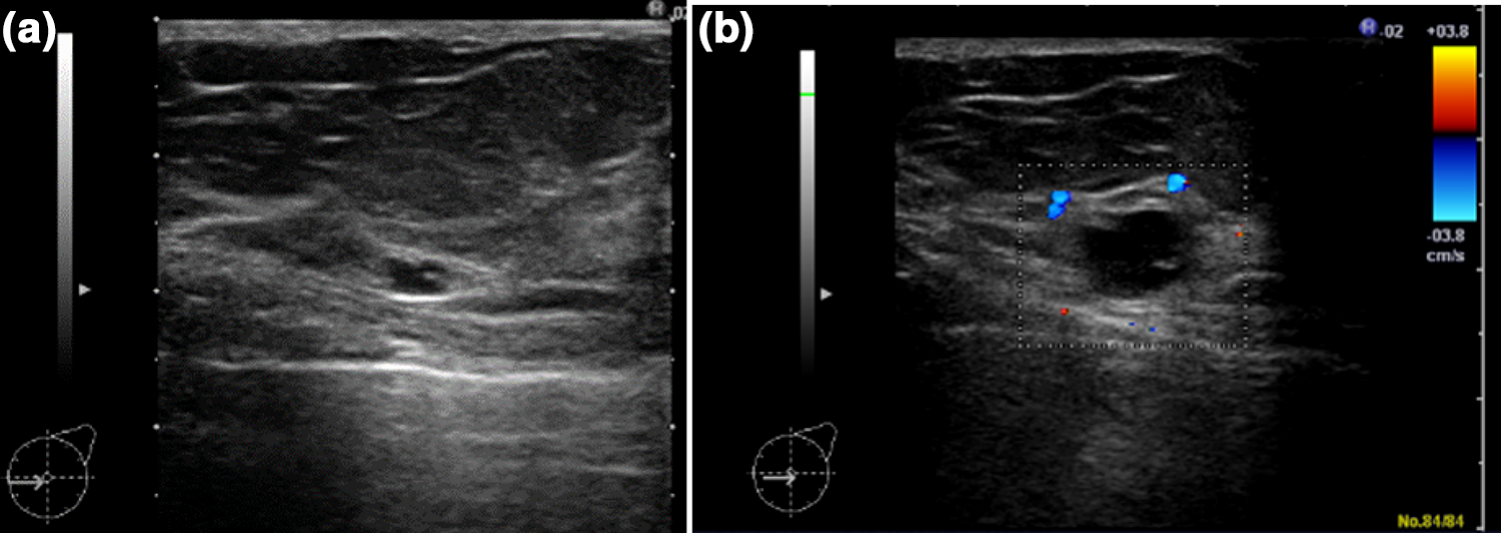
**a** This image showed the cystic pattern and further examination was considered unnecessary in 2011. **b** This lesion was diagnosed as microinvasive cancer (0.5 mm) in 2012

This pattern was noted in 6512 cases. These were essentially Category 2 and benign [4], but one case (0.015%) was malignant. The malignant case is shown in Fig. 2. It showed the cystic pattern and further examination was considered unnecessary, but when the patient underwent opportunistic screening after one year, an intracystic tumor with a slight internal echo was detected and subjected to further examination, and it was diagnosed as microinvasive cancer (0.5 mm) (Fig. 2).

### Mixed pattern

Ninety cases of masses visualized as an intracystic tumor were assessed as Category 3 and subjected to further examination. According to the guideline, masses showing the mixed pattern with an entire mass size of 5 mm or smaller are assessed as Category 2, for which further examination is unnecessary. Three cases corresponded to this classification. One patient assessed as Category 3 rejected further examination, and a conclusion was not reached in this case. No definite diagnosis of cancer could be made in any of the 90 Category 3 and three Category 2 cases (93 cases in total).

### Solid pattern with obvious benign features

A. Well-defined and smooth tumor with a very low depth width ratio, less than 2 cm in diameter.

**Fig. 3:**
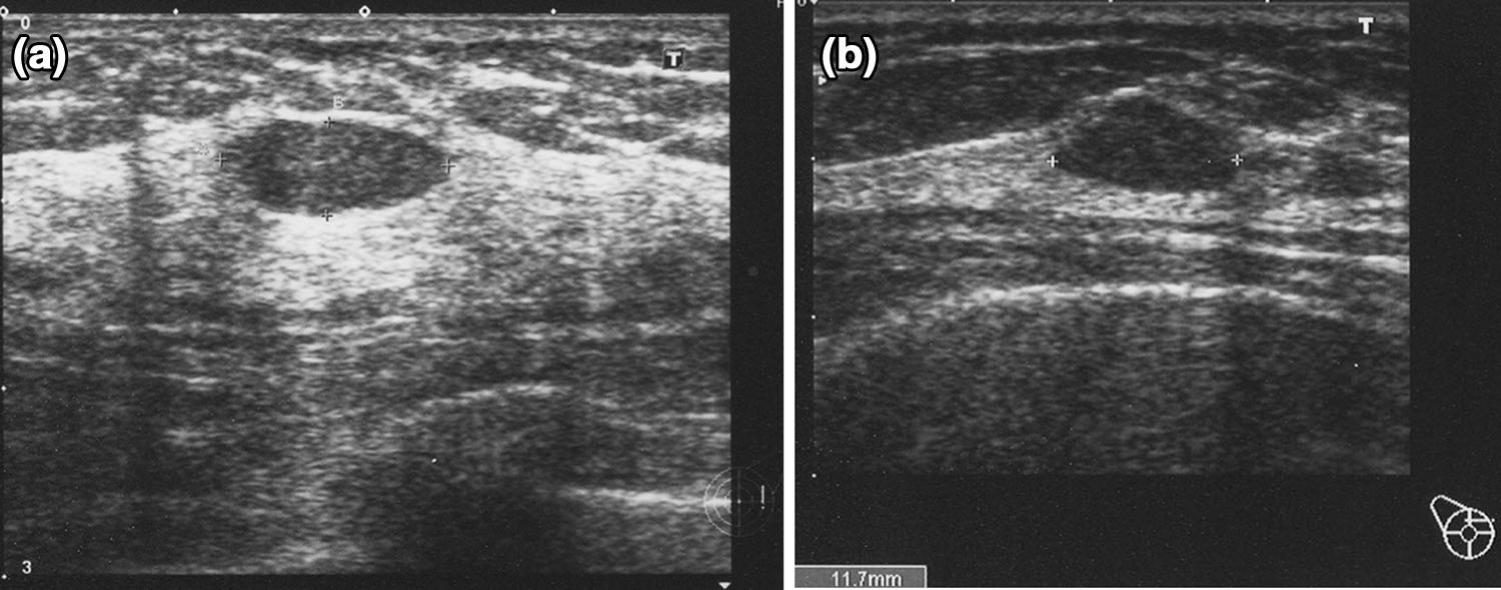
**a** This case was evaluated as fibroadenoma at the initial screening, but it was re-evaluated on screening 2 years later and was diagnosed as 1-cm luminal-type invasive ductal carcinoma (IDC). This image was at the initial examination. **b** This case was also evaluated as fibroadenoma at the initial screening, but the mass had grown to luminal-type IDC with a 5-cm invasion diameter 3 years later. This image was at the initial examination

These findings are assessed as Category 2, being considered typical fibroadenoma, for which further examination is unnecessary [5]. Two of 1,431 cases were malignant, accounting for 0.14% (Fig. 3a, b). The negative predictive value was 98.6%. One case was detected on screening 2 years later, and the 1-cm mass was luminal-type invasive ductal carcinoma (IDC). The other patient underwent screening 3 years later, and the mass had grown to luminal-type IDC with a 5-cm invasion diameter.

B. Mass with coarse calcification.

An old fibroadenoma accompanied by typical calcification is assessed in this box. It is Category 2, not requiring further examination in the diagnostic tree [5]. None of the 52 cases was malignant. The negative predictive value was 100%.

C. Mass with anterior curvilinear high echoes and absence or attenuation of posterior echoes.

This ultrasonographic image suggests a typical complicated cyst [6]. Seventy cases corresponded to this and none of them was malignant. The negative predictive value was 100%.

### Solid pattern with obvious malignant features

Masses with an echogenic halo [7] or interruption of the interface between adipose tissue and gland [8] are classified as this pattern. These findings suggest distinct invasion, and malignant disease is first considered, being assessed as Category 4 or 5. Twenty-two cases corresponded to this and 17 of them were malignant. The positive predictive value was 77.3%.

### Mass with multiple echogenic foci

This finding corresponds to microcalcifications on mammography. Even though no echogenic halo or interruption of the interface between adipose tissue and gland is observed, when the mass clearly contains echogenic foci on US, malignancy should be considered, being assessed as Category 4 or 5. Five of the 11 cases were malignant and the positive predictive value was 45.5%.

### Mass evaluated based on the maximum diameter and depth/width (D/W) ratio

Masses not showing any of the above features are classified based on the maximum diameter and D/W ratio. The results are shown. Setting the cut-off value of the D/W ratio at 0.7 [9–11], benignity and malignancy are considered when the value is below 0.7 and 0.7 or higher, respectively.

A. Mass with a 5 mm or smaller diameter.

**Fig. 4:**
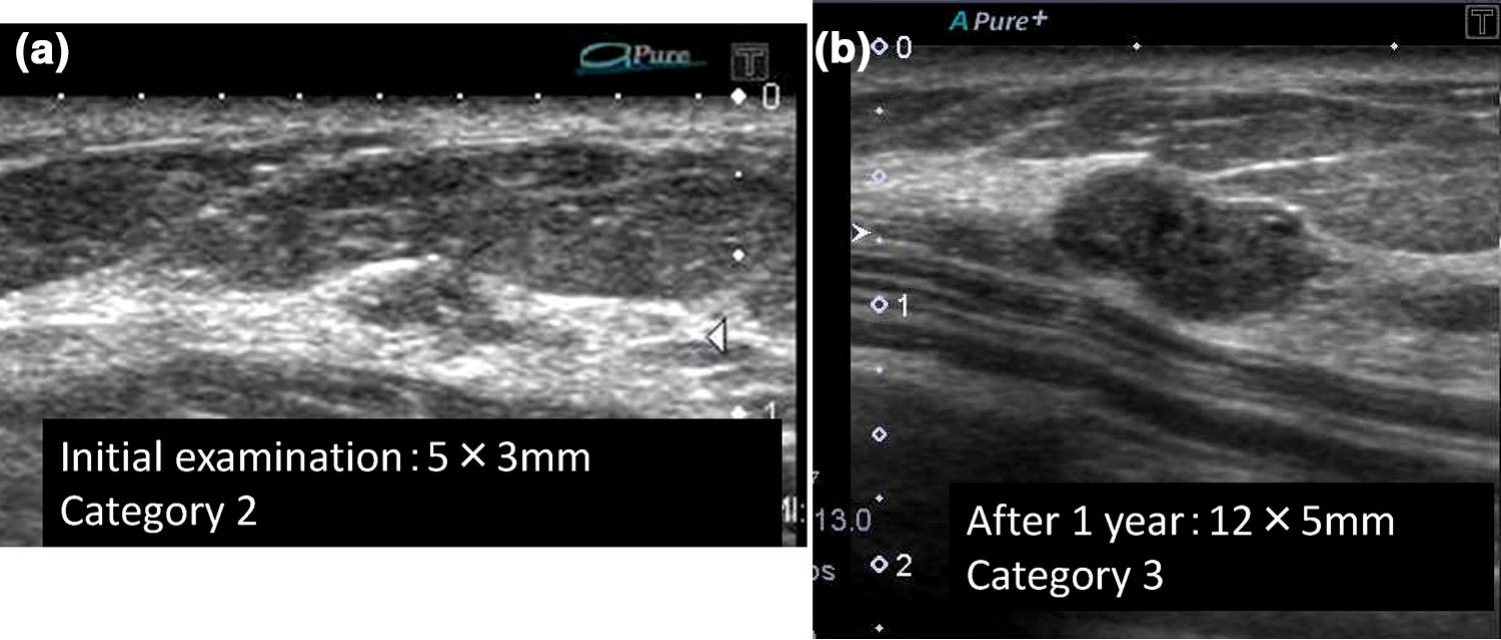
**a** The size of this lesion was 5 × 3 mm at the initial screening. In addition, there were lots of similar findings. This lesion was considered Category 2, requiring no further examination. **b** This patient underwent opportunistic screening after one year, and a 12-mm mass was detected and subjected to further examination. The mass was luminal-type mucinous carcinoma

**Fig. 5:**
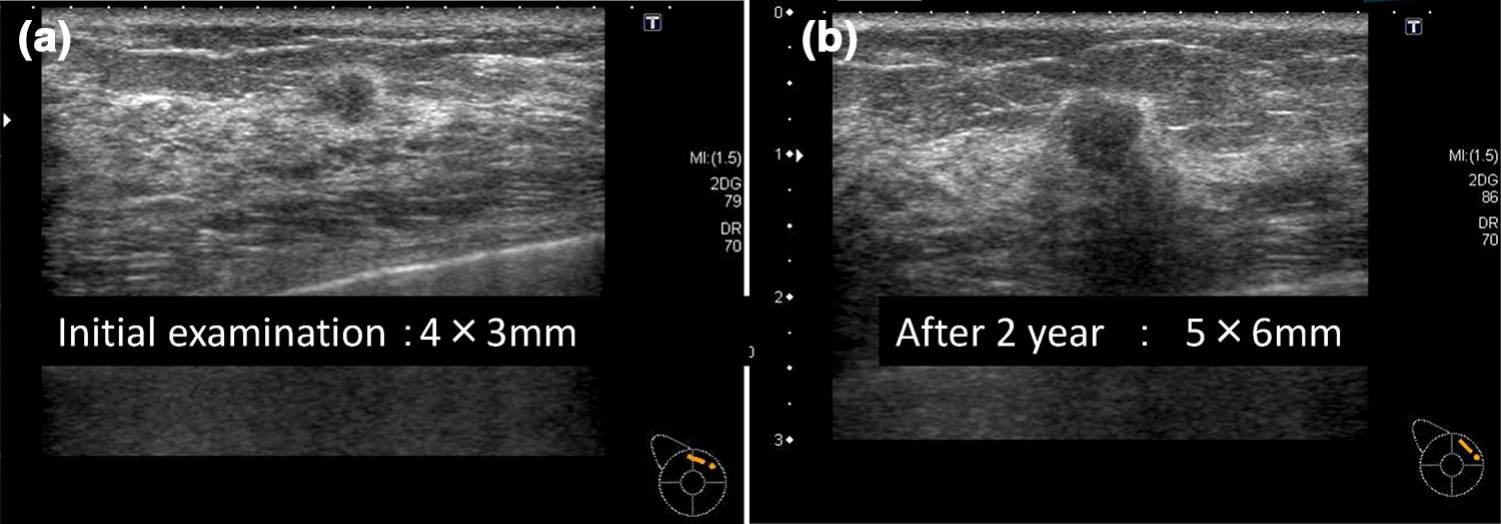
**a** The size of this lesion was 4 × 3 mm at the initial screening. This lesion was considered Category 2, requiring no further examination. **b** The lesion grew to luminal-type IDC with a 5-mm invasion diameter 2 years later

Masses with a 5 mm or smaller diameter are divided into those with a D/W ratio below 0.7 and 0.7 or higher, but basically both are Category 2 requiring no further examination. Masses are assessed as Category 3 or higher only when further examination is strongly considered necessary due to an irregular shape. There were 501 cases with a D/W ratio lower than 0.7, and all cases were assessed as Category 2, but malignancy was identified in one of them (1/501, 0.19%). This patient underwent opportunistic screening after one year, and a 9-mm mass was detected and subjected to further examination. The mass was luminal-type mucinous carcinoma (Fig. 4). The DW ratio was 0.7 or higher in 406 cases: 397 cases were Category 2, and nine cases were assessed as Category 3 due to an irregular shape. No cancer was detected in the cases assessed as Category 2. Cancer was detected in four of the nine cases assessed as Category 3. Three cases were luminal-type invasive cancer, and the invasion diameters were 4 mm, 5 mm (Fig. 5), and 9 mm, respectively. One case was triple-negative (TN) breast cancer with a 4-mm invasion diameter. Of 406 cases of 5-mm or smaller mass with a D/W ratio of 0.7 or higher, four cases were breast cancer, accounting for 0.98%. Regarding the number of Category 3 cases (9) as the parameter, the cancer detection rate was 44.4%.

B. Mass with a diameter of 5–10 mm and a D/W ratio lower than 0.7.

**Fig. 6:**
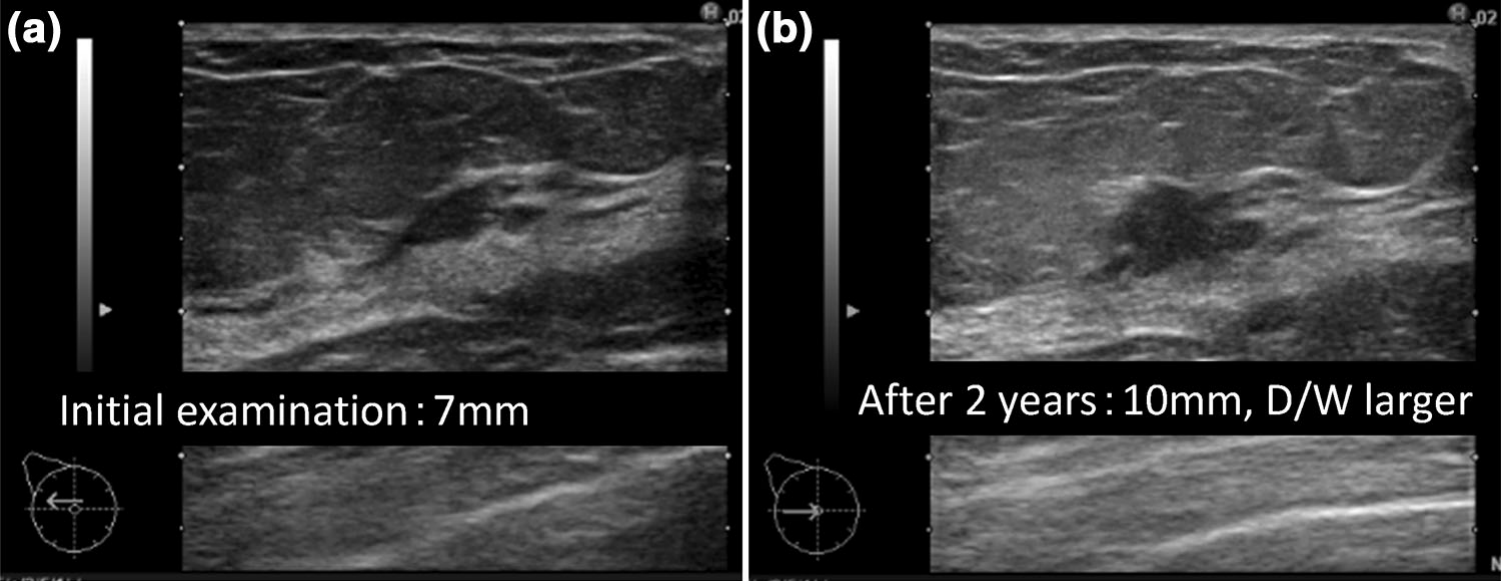
**a** The size of this lesion was 4 × 3 mm with a D/W ratio lower than 0.7 at the initial screening. This lesion was considered Category 2, requiring no further examination. **b** This lesion was detected by screening after 2 years and subjected to further examination, and it was TN breast cancer with a 1.5-cm invasion diameter

Masses with a D/W ratio lower than 0.7 are basically Category 2, requiring no further examination. Four of the 979 cases were cancer, and the cancer discovery rate was 0.4%. Three of them were breast cancer detected by screening after 2 years and subjected to further examination, and these were DCIS, 1.4-cm luminal-type invasive ductal carcinoma, and 1.5-cm TN breast cancer, respectively (Fig. 6). In the remaining case, the opposite side was assessed as Category 3 and subjected to further examination, and it was 6-mm TN breast cancer detected on further examination.

C. Mass with a diameter exceeding 5 mm up to 10 mm and a D/W ratio of 0.7 or higher.

Masses classified as this are basically Category 3 or higher. Eleven of the 162 cases were malignant and the cancer discovery rate was 6.8%.

D. Mass with a diameter exceeding 10 mm and a D/W ratio lower than 0.7.

Lesions with these findings are subjected to further examination regardless of the D/W ratio. The D/W ratio was lower than 0.7 in 233 of all cases, eight of which were malignant, and the cancer discovery rate was 3.43%. The D/W ratio was 0.7 or higher in 38 of all cases, six of which were malignant, and the cancer discovery rate was 15.8%.

## Discussion

Two types of cancer screening are performed in Japan. One is a population-based screening and the other is an opportunistic screening. Mammography has been carried out in the population-based screening as a screening method with evidence of decreasing mortality rate. On the other hand, ultrasonographic screening is already being performed as opportunistic screening in many screening centers or institutes, although its usefulness has not been proven.

Recall criteria for ultrasonographic screening were described in 2014 in the 1st edition based on consensus among experts, and it was revised in the 3rd edition in 2016.

The purpose of this study was to validate the usefulness of the recall criteria in multiple centers by collecting data from five institutions in various regions of Japan.

First, we investigated the validity of the findings regarded as Category 2, requiring no further examination in the flow chart.

In the box for the cystic pattern, 6512 lesions were confirmed, one of which was malignant. This case was subjected to further examination on screening one year later and confirmed to be microinvasive cancer, but it may not be related to the vital prognosis because the invasion diameter was 0.5 cm. Since the frequency of cysts is very high in screening, it is obvious that a rise in the recall rate will result if cysts are assessed as Category 3 or higher and need further examination. Therefore, it is very important to educate and ensure that cysts are always classified as Category 2, requiring no further examination even when detected on opportunistic screening, such as during a medical check-up.

Findings that can be concluded to be benign and Category 2 requiring no further examination are three features of the solid pattern. There were 1431 lesions smaller than 2 cm with a sufficiently low D/W ratio and a circumferentially clear and smooth boundary, for which typical myxoedematous fibroadenoma was considered, and two cases were cancer (Fig. 3a, b). This may suggest that very few cancer cases are included in this classification. In addition, one of the two cases of breast cancer was 1-cm luminal breast cancer detected on screening 2 years later, which may be within the acceptable range. The other case was detected as 5-cm triple-negative (TN) breast cancer 3 years later. This could have been detected earlier if the patient had undergone screening every 2 years. Looking retrospectively at the image from the first examination in this patient, the D/W ratio was slightly high. To prevent overlooking highly malignant TN breast cancer in low-echo masses with a clear boundary, the accuracy may be increased by thoroughly assessing lesions with a D/W ratio of 0.5 or lower as those with a “sufficiently low D/W ratio”. Fibroadenoma is a benign mammary gland disease with a high incidence, for which accurate evaluation of this feature is very important. The D/W ratio should be accurately assessed by ingeniously utilizing the diagnostic tree so that further examination of typical fibroadenoma cases can be avoided. But more importantly, there is a limit to cancer detection, and the purpose of these criteria is not to detect all breast cancers. The cancer screening professional should understand that these criteria may fail to detect rare breast cancer that may grow rapidly.

The 3rd type of obviously benign solid mass is those with anterior curvilinear high echoes and absence or attenuation of posterior echoes, which is a typical complicated cyst with a high incidence. No malignant case was included in the 70 cases of complicated cyst, which may be a significant finding.

The above criteria were mostly valid in terms of concluding that the mass was benign and did not require further examination, although a few cases of breast cancer were included.

How about the results of typical malignant findings? Findings indicating malignancy include an echogenic halo and interruption of the interface between adipose tissue and gland. These two findings are considered important features reflecting invasion. The positive predictive values of these features were higher than 95% in a multicenter cooperative study performed by JABTS, showing their high reliability. The positive predictive value was also high (77.3%) in our study. The mass contained multiple echogenic foci in five of 11 cases, showing a high malignancy rate (45.5%). Only 11 cases were included in this classification. When a mass shows an echogenic halo or interruption of the interface between adipose tissue and gland, being likely to be malignant, the category of the mass is already evaluated based on the feature, and this may have been the main reason for the small number of cases. The problem may be slight variation in detection of echogenic foci among devices and physicians, but further investigation of these features may be necessary because the number of cases was small.

Let’s move on to findings where it was difficult to determine whether the mass was benign or malignant, including Category 3.

First, there were 90 cases of the mixed pattern at all institutions combined, but none of them could be definitely diagnosed as malignant. One case was suspected of being apocrine DCIS on needle biopsy, but it could not be definitely diagnosed, and a final diagnosis was not made because the patient rejected further examination. If this case was cancer, it was very likely to be noninvasive carcinoma. The mixed pattern is mainly assumed to be an intracystic tumor. Accordingly, even though the mass is an intracystic tumor and malignant, most cases are likely to be noninvasive ductal carcinoma or microinvasive cancer. Subjective symptoms are relatively likely to develop in intracystic papilloma and intracystic cancer. Considering that there were almost no cases of malignant disease, this mixed pattern is assessed as Category 3 at present, but it should be re-evaluated as Category 2 requiring no further examination in the next revision.

Regarding the final classification based on the maximum diameter and D/W ratio, cancer was discovered in only one of 501 cases of 5-mm or smaller masses with a D/W ratio lower than 0.7 (Fig. 4), and this cancer was 12-mm luminal-type mucinous cancer detected on screening one year later. Based on the frequency and pathology of the breast cancer detected, it was considered reasonable to be assessed as Category 2. There were 406 cases of 5-mm or smaller masses with a D/W ratio of 0.7 or higher, 397 of which were assessed as Category 2, and no breast cancer was included in these. Nine cases were assessed as Category 3 because of an irregular shape. Four of them were breast cancer, and the positive predictive value was 44.4%, which was higher than expected. The cancer was 1-cm luminal breast cancer in three and 4-mm TN breast cancer in one.

The important point is how to reduce the rate of assessing masses as Category 3 based on the irregular shape in the case of masses with a 5-mm or smaller size and a D/W ratio of 0.7 or higher, which should be basically assessed as Category 2, to narrow down cases for which breast cancer should truly and strongly be considered. Only nine of the 406 cases were assessed as Category 3, accounting for only 2.2%. The specificity must not be reduced by easily evaluating masses as Category 3 in this step out of excessive concern for overlooking cancer. However, if cases to be assessed as Category 3 can be carefully selected and subjected to further examination, assessment using these criteria may effectively work. Further investigation with an increased number of cases from different institutions may be essential.

Regarding masses with a size exceeding 5 mm but not greater than 10 mm and a D/W ratio lower than 0.7, 975 of 979 cases were benign lesions, and the negative predictive value was very high (99.6%). Three cases of breast cancer and one case of simultaneous bilateral TN breast cancer were detected 2 years later (Fig. 6), but the results may be valid.

Masses with a size exceeding 5 mm but not greater than 10 mm and a D/W ratio of 0.7 or higher and those larger than 10 mm with a D/W ratio below 0.7 and of 0.7 or higher are currently assessed as Category 3 or higher in the recall criteria. The cancer detection rates were 6.8% (11/162), 3.4% (8/233), and 15.8% (6/38), respectively. It is desirable to further narrow down cases to be subjected to further examination using dynamic testing and increase the positive predictive value, for which further investigation is needed.

There were several problems with verification of the flow chart involving actually screened cases. First, the study period, age, and screening conditions varied among the five institutions. However, considering that opportunistic screening is performed under various conditions in Japan, the results collected under different conditions may be valid as study data. Although the conditions are different, the accuracy of sonographers, physicians, and devices is controlled in all institutions. The study was initiated within 5 years, and this may not be a major problem. Secondly, at present, ultrasonography is not basically used in opportunistic screening in Japan, and the subjects examined in this study were mostly younger than 40 years old, being in their 30 s, and females in their 20 s were also included. Their age was different from that with a high incidence of breast cancer, and this may have interfered with correct verification. Although there is no radiation exposure, unlike mammography, thoughtlessly involving young women has to be avoided. To further verify the flow chart to establish better recall criteria, it is necessary to verify data collected from women in their 40–50 s, at which the incidence of breast cancer is high in Japanese. Thirdly, overdiagnosis, which is considered the first on the list of disadvantages of screening [12–15], was not investigated. The problem with overdiagnosis cannot be approached by only evaluating the positive predictive value of subjecting Category 3 or higher cases to further examination. It has been reported that many cases of breast cancer detected by ultrasonography are less malignant luminal-type breast cancer [16]. It may be necessary to investigate the subtype of detected breast cancer in multiple institutions.

## Conclusion

The screening flow chart in the JABTS guidelines was verified in cases with mass at multiple institutions. The current category classification was mostly valid, but further verification and revision may be necessary.
